# Metal accumulation sites in fruits

**DOI:** 10.1101/2023.12.12.571343

**Published:** 2025-08-28

**Authors:** Kumbirai Deon Mandebere, Utku Deniz, Katarina Vogel Mikus, Cagdas Son, Seckin Eroglu

**Affiliations:** 1 Department of Biological Sciences, Middle East Technical University, Ankara, Turkey; 2 Jozef Stefan, Jamova cesta 39, Ljubljana, Slovenia; 3 Biotechnical Faculty, University of Ljubljana, Jamnikarjeva ulica 101, Ljubljana, Slovenia

**Keywords:** fruit, synchrotron, X-ray, metal, iron, MTP8, germination, tomato

## Abstract

Global fruit production suffers from pre- and post- harvest losses, part of which are related to metal deficiencies. Despite fruits being one of the most widely consumed plant parts, the spatial distribution of metals and their possible physiological impact remained largely unexplored. In this study, we searched for conserved metal accumulation sites in fruits of various crops and investigated their physiological function. By using X-ray and histochemical-based techniques, we found that calcium accumulated in the outermost hardened tissues, potassium in fleshy tissues, and iron (Fe) in vascular tissues. Furthermore, seeds accumulated Fe in the fruit-seed juncture, which remained in the chalazal part of the seed upon dispersal. In *Arabidopsis thaliana*, seed chalazal Fe formed a distinct circular shape, which appeared diffuse in *metal tolerance protein 8* mutants. Seed chalazal Fe appeared also in tomato seeds, albeit more diffused and at higher concentrations. In tomato, treatment with the Fe immobilizer desferoxamine delayed germination in wild-type plants but not in mutants with low chalazal Fe, indicating that chalazal Fe may contribute to germination. This study marks the first systematic application of X-ray fluorescence and histochemistry to investigate conserved metal localization sites in mature fleshy fruits. The discovery of metal hotspots in fruits opens new research avenues to understand fruit physiology, which may, in the long run, contribute to food security.

## Introduction

Fruits are essential to a nutritious human diet. To meet and sustain the dietary needs of the growing global population, fruit production must increase from one billion tons to over two billion tons per year by 2050 (Stratton et al., 2021; Willett et al., 2019). A key challenge in achieving this goal is reducing pre- and post-harvest losses, which can account for up to half of the production, making fruits the most wasted type of food (Gustavsson et al., 2011; Wang et al., 2022). Metal deficiencies are significant factors contributing to such losses, limiting the quality and quantity of fruits (Andresen et al., 2018; Kaya and Higgs, 2002; Suzuki et al., 2003). The effects of metal deficiencies can be either direct or indirect. As a direct effect, insufficient Ca levels in apples can result in bitter pit disease (Song et al., 2018; Torres et al., 2021; Van Goor, 1971). As an indirect (i.e., systemic) effect, the lack of a particular metal in sink tissues can cause metabolic remodeling, leading to lower quantity and quality of fruits (Àlvarez-Fernàndez et al., 2006; Álvarez-Fernández et al., 2003; El-Gioushy et al., 2021). Furthermore, metal deficiencies may also weaken the defense of fruits against several microbial diseases (Conway et al., 2001; Morina and Küpper, 2022).

Despite the importance of metals in fruit biology, protocols for the investigation of their distribution in plant tissues do not always lead to the desired results. The primary methods for exploring the spatial localization of metals in plant organs such as seeds, roots, or leaves are X-ray-based techniques (Mijovilovich et al., 2020; Wu and Becker, 2012) and histochemical staining. However, these methods are often impractical for fleshy fruits with high water content (Lombi et al., 2011; Yadav et al., 2021; Zhao et al., 2014), which complicates the use of many sample preparation techniques. For example, fixatives may not effectively replace water, the physical structure of fruits may be undermined by water loss, and slicing of water-filled soft tissues into uniform sections may be challenging and require specialized expertise. Consequently, spatial metal analysis of fruits is currently limited to non-fleshy varieties such as grains or pods and, with only a few exceptions, does not include juicy, fleshy fruits like tomatoes or oranges (Trebolazabala et al., 2017). At present, researchers collect samples from various parts of the fruit and compare their total metal concentrations, resulting in limited spatial information (Kalcsits, 2016; Ramesh et al., 2021; Saquet et al., 2019). Therefore, partially due to inefficient protocols, our understanding of how metals localize in fruits and the physiological functions they perform remains limited.

Here, we systematically investigated metal accumulation sites in freeze-dried fruit samples using X-ray fluorescence and histochemical staining. This approach identified metal “hot spots”, some of which are surprisingly well-conserved. We further studied one of these metal hotspots to investigate its possible physiological functions.

## Material and Methods

### Research material

Fruits of various species were purchased from local markets and bazaars in Turkey throughout the year (Table S1). For experiments with tomato seeds, if not otherwise indicated, wild-type *Solanum lycopersicum* cv. Bonner Beste plants and its mutant *chloronerva* were used (Herbik et al., 1996). All Arabidopsis lines used in this study were derived from the Col-0 ecotype. The *mtp8–1* and *mtp8–2* mutants have been characterized before (Eroglu et al., 2016). The SUC2::GFP line was a donation and has been published elsewhere (Kalmbach and Helariutta, 2019).

### Tabletop XRF

To prepare the fruits for XRF analysis, they were freshly sliced both longitudinally and cross-sectionally to a thickness of 2–3 mm using a V-blade (Figure S1). The prepared fruit slices were immediately immersed in liquid nitrogen and kept frozen at −80°C for 24 hours. Frozen samples were then placed in a vacuum freeze dryer for a main drying phase of 23 hours at −54°C and 0.024 millibars, followed by an additional hour of final drying at −60°C and 0.011 millibars. Once dried, fruit samples were stored at room temperature until the analysis.

Freeze-dried fruit samples were analyzed by a micro XRF analyzer (Horiba XGT-9000, Japan). The benchtop system was equipped with a Rh tube (50 kV, 1 mA) yielding a polychromatic excitation beam focused to 15 μm by a polycapillary lense. X-ray fluorescence was detected with a silicon drift diode (SDD) detector. The samples were mapped with 100–120 μm step size and 200 ms dwell time. The spectra in each pixel were recorded with the Horiba software, exported as .H5 files, and processed with the PyMCA 5.8.1 toolkit (Bolte et al., 2011) where full spectra deconvolution and fitting was performed. Maps were then generated in the RGB correlator of PyMCA on the basis of the intensities of particular fluorescence K lines (Solé et al., 2007).

### Synchrotron XRF

For examining metal enrichment in the vasculature of tomato fruits (*Solanum lycopersicum* cv. Moneymaker) in high resolution and with minimum treatments, fresh cut tomato pieces were embedded in the cryo-embedding matrix OCT and immediately plunged into liquid nitrogen. Fifty μm cross sections were obtained using a Leica RM2265 semi-automated rotary cryomicrotome and immediately transferred to a sample holder between two Ultralene foils. The samples were kept in liquid nitrogen during the transfer and analysis with the Scanning X-ray Microscope (SXM) at ID21 X-Ray Microscopy Beamline at the European Synchrotron Radiation Facility (Grenoble, France). The beam size was focused using Kirckpatrick Baez mirror optics to about 1×0.6 (HxV) μm^2^. Elemental localization and distribution of Fe were performed at 7.3 keV excitations above the Fe K-edge and 100 ms dwell time. The energy was selected using a double-crystal monochromator with a Si111 crystal pair. The X-ray fluorescence signal from the samples was detected using a silicium drift diode detector with an 80 mm^2^ active area from SGX Sensortech. The incoming beam intensity was monitored using a photodiode. The X-ray fluorescence data from each pixel in the maps were fitted to obtain the final elemental distribution images using the batch processing routines from PyMCA 5.9.2.

For examining metal accumulation in the chalaza of tomato seeds in high resolution, two-dimensional SXRF imaging was conducted. Dry seeds were placed on a Kapton tape. Images were collected at beamline 4-BM of the National Synchrotron Light Source-II (NSLS-II; Brookhaven National Laboratories, Upton, NY, USA). The elemental maps were collected using an incident energy of 12 keV, a 7μm step size, and a 100 ms dwell time. Raw data from the NSLS-II was analyzed using the Larch software package to produce fitted X-ray fluorescence images. Final SXRF images and intensity profile plots were generated using the Nikon NIS-elements software.

### Histochemical staining of fruits

For preparing the fruit samples for histochemical staining, freeze-dried fruit pieces that were prepared as described above for the tabletop XRF analyses were used as starting material. Colorful fruits were subjected to decolorization. Decolorization was conducted by immersing the pieces into a fixing solution (methanol: chloroform: glacial acetic acid; 6:3:1) in a glass Petri dish and shaking at 90 rpm until the natural color disappeared. The fixing solution was renewed several times during the shaking. To stop the decolorization step, the fixative was removed by washing the samples at least three times with distilled water.

For the histochemical staining of Fe, Perls staining was used as previously described (Roschzttardtz et al., 2009). Briefly, Perls stain solution was prepared fresh by mixing 4% K-ferrocyanide (K_4_Fe(Cn)_6_) and 4% HCl stock solutions in a 1:1 (v:v) proportion and vacuum infiltration for one hour at 500 millibars. Next, the staining solution was removed, and the samples were washed three times with distilled water for 1–2 min each by slight shaking. The samples were stored in distilled water at 4°C until observation with a stereo microscope.

For histochemical Fe staining on the thin cross sections of tomato fruits, samples prepared for Perls staining were first embedded in resin, cut, and then stained. Pieces were washed three times with 0.1 M phosphate buffer (pH 7.4) and dehydrated in successive baths of 50%, 70%, 90%, 95%, and 100% ethanol, butanol/ethanol 1:1 (v/v), and 100% butanol. Then, the pieces were embedded in Technovit 7100 resin (Kulzer, Tokyo, Japan) according to the manufacturer’s instructions and sliced into thin sections (10 *μ*m). The sections were deposited on glass slides and incubated for 45 min in Perls stain solution. Perls solution was removed, slides were washed three times and directly observed with a compound microscope.

### Histochemical staining of Arabidopsis seeds

To observe whether Fe also accumulates in the seed chalaza of *Arabidopsis thaliana*, seeds were prepared as illustrated (Fig. 5). Histochemical staining of Arabidopsis seed parts was conducted as described above followed by DAB intensification. For DAB intensification, the stained seed parts were incubated for 30 min in a 0.1 M phosphate buffer (pH 7.4) solution containing 0.025% (w/v) DAB, 0.005% (v/v) H_2_O_2_, and 0.005% (w/v) CoCl_2_ (Roschzttardtz et al., 2009). The reaction was stopped by rinsing the samples with distilled water. Images were captured with a compound microscope.

### Elemental analysis by ICP-OES

To quantify Fe accumulation in the chalazal part, elemental analysis on seed parts were conducted. To this end, mature tomato seeds (*Solanum lycopersicum* cv. Candela) were imbibed for 24 hours and dissected into three parts as shown in Figure 2. Dissected samples were dried in an oven at 65°C for one week. Dried plant samples were finely ground with an electric coffee grinder. Approximately 0.3 g of the dried and ground plant samples were weighed and placed in microwave digestion tubes. On top of each sample, 2 ml of 30% H_2_O_2_ and 5 ml of HNO_3_ were added, and the digestion was conducted in a closed-vessel microwave system (MarsExpress; CEM Corp., Matthews, NC, USA). After cooling down sufficiently, the total sample volume was finalized to 20 ml by adding double-deionized water, and samples were filtered through analytical filter papers (Macherey-Nagel, Ø125 mm, blue band). Inductively coupled plasma optical emission spectrometry (ICP-OES) Agilent 5800 ICP-OES (Agilent Technologies, USA), was used to determine the concentrations of micronutrients in digested plant samples. The accuracy of the element analyses was validated using certified standard reference materials obtained from the National Institute of Standards and Technology (Gaithersburg, MD, USA).

### Ferric chelate reductase assay

Ferric chelate reductase activity of endosperm exudates was assessed according to a method previously described (Grillet et al., 2014). Mature tomato seeds (*Solanum lycopersicum* cv. Candela) were imbibed in water for 24 h at room temperature and cut longitudinally. Embryos were discarded, and five endosperms were pooled as one biological sample (n=3). Isolated endosperms were immersed in distilled water and allowed to release their exudates for 24 hours. The obtained exudate solution was then mixed (1:2 ratio) with ferric reductase activity assay solution (0.2 mM CaSO4, 5 mM MES (pH 5.5), 0.2 mM ferrozine, and 0.1 mM Fe-EDTA) on a 12-well plate in the presence or absence of ascorbate oxidase enzyme (1.5 AOX units ml^−1^). Following an incubation of 3 hours, endosperms were quickly dried on tissue paper and weighed. An 800 μl aliquot was taken from the remaining solution and the absorbance was measured at 562 nm. Experiments were repeated three times with at least three biological replicates and the result of one representative experiment is shown.

### Confocal microscopy

To observe phloem remnants in mature Arabidopsis seeds, the well-known phloem marker SUC2::GFP was used (Kalmbach and Helariutta, 2019). Arabidopsis seeds expressing the SUC2 construct were imbibed in distilled water for 24 hours at 4°C. After imbibition, the seeds were dissected and chalazal+micropylar parts were excised. These pieces were imaged using a confocal laser scanning microscope (Leica DMI4000 B; Leica Microsystems GmbH, Wetzlar, Germany) equipped with a 488-nm GFP filter. The images were subsequently overlaid using the Fiji (ImageJ) imaging processing package.

### Germination tests

To determine how Fe availability impacts germination, germination tests were conducted with tomato (*chloronerva* and its wild type Bonner Beste) in the presence and absence of a strong Fe chelator. Seeds were sown on Petri dishes (30 seeds per dish, three replicates) filled with water agar (0.7% agar, buffered at pH 5.6 with MES) with or without desferoxamine (250 μM). Plated tomato seeds were incubated for two days in a refrigerator for stratification and then placed in a growth chamber at 25 °C under continuous light. Germination was scored over time, with a seed considered germinated when the radicle protruded through the testa. The impact of desferoxamine on germination was calculated by subtracting the IC50 values of the best-fit curves of the control from those of the desferoxamine treatment.

## Results

### Ca, K, and Fe accumulate in specific tissues of the fruit

**Figure 1:**
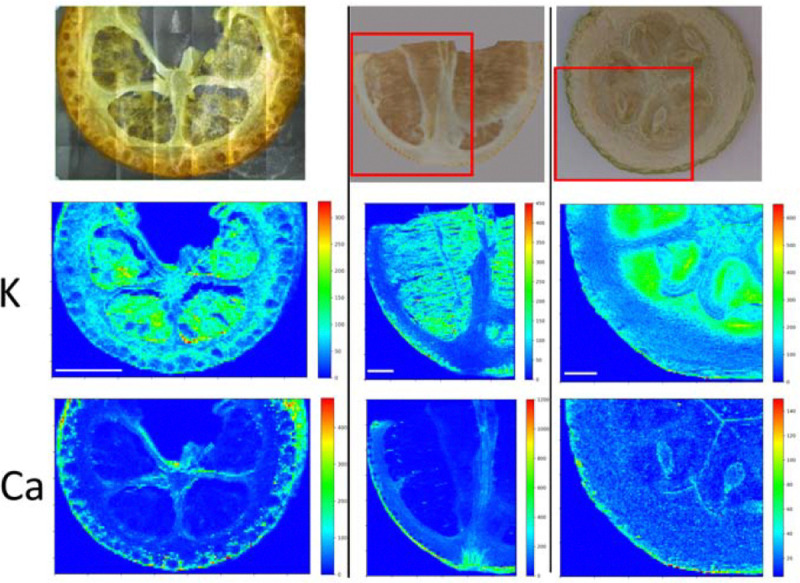
Calcium accumulates in epidermal and K in fleshy parenchyma tissues of fruits. Freeze-dried fruit slices were examined with a bench-top micro-XRF. Left, kumquat; middle, lemon; right, squash. Red rectangles denote the analyzed area. At least two independent pieces were observed for each fruit with comparable results. Scale bar is 5 mm. K: Potassium, Ca: Ca.

**Figure 2:**
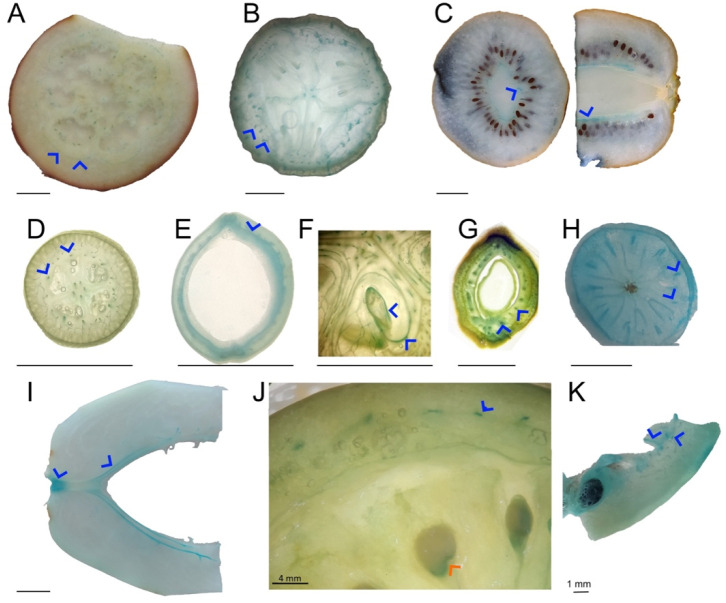
Iron accumulates in the fruit vasculature of diverse species and in the fruit-seed juncture in tomato. Cross-sectional or longitudinal cut fruit pieces were freeze-dried and decolorized with fixatives (methanol: chloroform: glacial acetic acid; 6:3:1). After fixation, the pieces were submerged in Perls staining solution for 2 to 16 hours. Blue coloration indicates Fe accumulation, blue arrowheads highlight vasculature stained blue in different fruit species. For this figure, images of fruits with noticeable Fe accumulation in the vascular bundles were chosen. Note that tomato accumulated Fe in the fruit seed juncture, shown by a red arrowhead. A-K; eggplant, cucumber, kiwi, kumquat, haricot, squash, green almond, blueberry, papaya, tomato, and passion fruit. At least five pieces of fruits were investigated, and a representative sample was photographed. Bar scale is 1 cm, unless otherwise indicated.

By introducing an initial freeze-drying step, we determined metal localizations in fruit pieces obtained from various crop species using a bench-top X-ray machine. We opted for fruits from crop species–including tomato, okra, black grape, blueberry, lemon, aubergine, kumquat, squash, and strawberry–due to their size, which is generally larger than those of wild plants. While X-ray mapping revealed metal localizations, it provided only limited resolution. Alternatively, histochemical stains have been used to localize metals, even in cellular compartments (Eroglu et al., 2019). We found that in all fruits K accumulated in their fleshy (water rich) parts (Fig. 1, Fig. S10). We also noticed that Ca preferentially accumulates on the outer skin of the fruits. To identify accumulation sites of Fe, we stained fruit slices with Prussian Blue (also known as Perls staining). Fruits showed either homogeneous staining (Fig. S2) or a more pronounced staining throughout their vascular tissues (blue arrow heads in Fig. 2). This result shows that in fruits, Fe is preferentially stored in the vasculature in a conserved manner. The use of fixatives has been criticized due to the possible remobilization of metals, leading to artifacts and misinterpretation of the results (van der Ent et al., 2018). To address this constraint, we employed synchrotron X-ray mapping, which allowed resolutions sufficient to map vasculature in fruit tissues. Mapping of freshly cut tomato pericarp under cryo conditions revealed a hollow Fe accumulation pattern, possibly corresponding to vasculature (Fig. S3). While we were unable to identify Fe-accumulating cell types, our result suggests that the preferential Fe accumulation in the vasculature is not an artifact due to sample preparation.

### Iron in the chalazal region of tomato seeds facilitates germination

**Figure 3:**
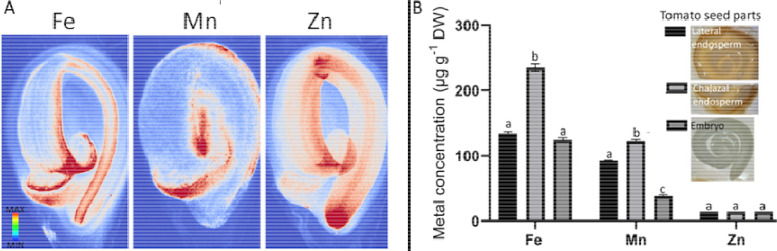
Metal loading to the fruit-seed juncture is specific to Fe and remains after seed dispersal. A, metal distribution in dry mature tomato seeds. Seeds were placed on Kapton tape and metal distribution was examined by synchrotron XRF. B, metal distribution in dissected seeds parts. Seeds were imbibed for one day and dissected to isolate embryos and lateral or chalazal endosperm. Seed parts were dried, and metal concentrations were determined using ICP-OES.

Seeds of various species have been extensively investigated for metal distribution (Eroglu et al., 2019; Ibeas et al., 2017); however, in tomato seeds, such distribution patterns remain elusive. In the present survey, we noticed Fe accumulation in the fruit-seed juncture of tomato slices (red arrowhead in Fig. 2). We examined whether metals other than Fe are also enriched in this region by mapping seed metal distribution using synchrotron X-ray fluorescence and ICP-OES. Mn partitioned towards the endosperm and Zn towards the cells that will develop into apical and root meristems (Fig. 3). Fe accumulated both in the embryo and the endosperm. While in the embryo, Fe accumulation was homogeneous, in the endosperm, Fe staining was enriched in regions close to the germination site. The chalazal endosperm (more accurately, chalazal and micropylar endosperm, including the associated seed coat) contained approximately twice as much Fe compared to the remainder of the seed. We further investigated whether this Fe accumulation is dependent on nicotianamine. Nicotianamine is a major chelator for metals including Fe in the phloem (Herbik et al., 1996). Seeds obtained from the nicotianamine-auxotroph mutant *chloronerva* showed lower concentrations of Fe, which, however, still accumulated preferentially in the chalaza (Fig. S4). These results indicate that Fe in the chalaza may play a specific role in tomato seed physiology.

In a germinating seed, endosperm-accumulated nutrients can be remobilized to support the embryo that is transforming into a seedling (Uraguchi and Fujiwara, 2011). To validate a possible role for Fe in feeding the germinating embryo, we examined the fate of the Fe deposits after germination. In seeds of one-week-old seedlings, seed endosperm tissues were degraded, reminiscent of nutrient remobilization from endosperm into the embryo (Fig. S5). However, Perls staining showed that these endosperm remnants preserved the preferential Fe accumulation in the chalaza (Fig. S5, denoted “After”) similar to the control (Fig. S5, denoted “Before”). Metal analysis showed that the Fe concentration doubled. Since the cultivation media did not contain nutrients, the observed increase in Fe concentrations was mostly due to loss of mass through endosperm degradation. These results negate the assumption that Fe in the chalazal nourishes the germinating embryo.

**Figure 4:**
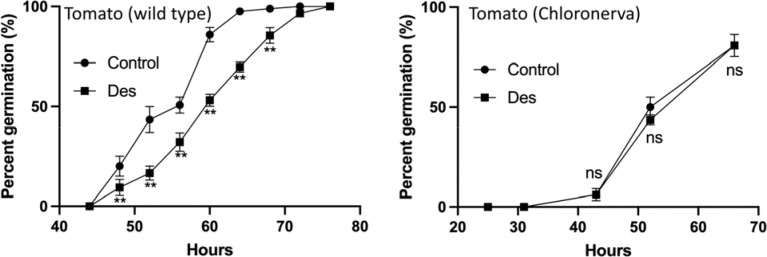
Iron loading in the fruit-seed juncture may facilitate germination. Left, desferoxamine, a strong chelator of Fe, decreases germination speed. Right, the impact of desferoxamine on germination is abolished in *chloronerva* mutants that contain low Fe concentrations. Tomato seeds were sown on water agar without (Control, circles) or with desferoxamine supplementation (Des, squares), and the germination percentage was calculated over time. The experiment was repeated four times with comparable results. Each treatment consisted of three plates, each containing thirty seeds. Error bars represent SEM. The asterisk indicates that the corresponding mean of the treatment is significantly different from the mean of controls for each time point indicated according to Student’s *t*-test (*P*<0.05).

Next, we assessed whether Fe could aid germination by comparing the effect of an Fe chelator on seeds with contrasting concentrations of Fe. The Fe chelator desferoxamine has been used to counteract Fe poisoning in humans (Rafati Rahimzadeh et al., 2023) and to render Fe stores in the plant tissues unavailable for biological processes (Aznar et al., 2014). Targeting Fe stores in seeds with desferoxamine led to a delay of approximately 6 hours (Fig. 4). However, the germination of the *chloronerva* mutant, which contains low concentrations of Fe, was not affected by desferoxamine, suggesting that Fe accumulation in the seed controls germination speed in tomato.

Germination speed is determined by the amount of hydroxyl radicals produced in the endosperm (Muller et al., 2009; Schweikert et al., 2002). A common pathway in nature for hydroxyl radical production is through the Fenton chain reaction, which requires Fe, hydrogen peroxide, and ascorbate (Burkitt and Gilbert, 1990a). Hydrogen peroxide production in tomato seeds during imbibition was shown before (Morohashi, 2002). We found that tomato endosperm also produces ascorbate during imbibition (Fig. S6). Taken together, it can be speculated that Fe aids germination by weakening the endosperm through the production of hydroxyl radicals.

### Chalazal Fe accumulation in Arabidopsis seeds follows remnants of vascular tissues and is dependent on MTP8

**Figure 5:**
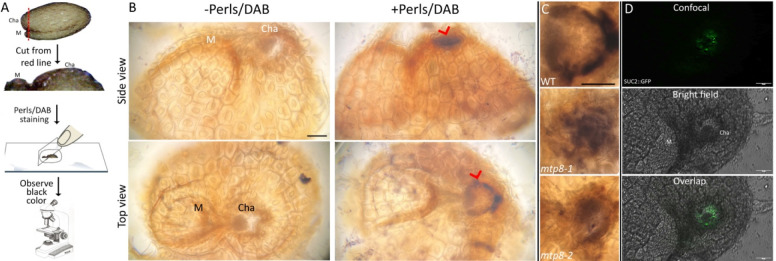
Iron accumulation in the fruit seed juncture is conserved in Arabidopsis seeds and determined by MTP8. A, sketch outlining the protocol. Seeds were cut along the red dashed line after one day of imbibition, stained with Perls/DAB and placed on a slide for examination under a light microscope. B, Perls/DAB staining of seeds and their unstained controls in side and top view. The ring-like black coloration indicates Fe accumulation. C, ring-like Fe accumulation in wild-type plants (WT) and *mtp8* mutants (*mtp8–1* and *mtp8–2).* These images are close-ups of the chalazal regions of the seeds. At least twenty seeds were analyzed for each genotype. M: micropylar region, Cha: chalazal region. -Perls/DAB: DAB intensification was conducted without Perls staining and serves as a negative control. D, GFP fluorescence of the phloem marker line SUC2::GFP. Mature seeds were imbibed for one day, cut following the sketch in Fig. 5A, and examined with a confocal laser microscope. At least five different seeds were investigated with comparable results. M: micropylar region, Cha: chalazal region. Bar: 50 μm

Next, we were interested in finding genetic components that mediate chalazal Fe accumulation. For this purpose, we employed the model plant Arabidopsis to profit from its genomic resources. We applied Perls staining with DAB amplification (Perls/DAB) to the cut seeds as depicted in Fig. 5A and investigated the black coloration representing Fe accumulation. Interestingly, Perls/DAB staining revealed Fe accumulation in the chalaza (Fig. 5B, top). In Arabidopsis, a chalazal Fe accumulation pattern has not been reported until now (Eroglu, 2018; Eroglu et al., 2017; Kim et al., 2006). However, unlike in tomato, this Fe accumulation was not evenly distributed but produced a dashed ring-like shape (Fig. 5B, bottom). We then screened Arabidopsis metal transporter mutants for disrupted chalazal Fe accumulation. Two independent alleles of *metal-tolerance-protein 8* (*mtp8*) knock-out lines showed a disrupted Fe accumulation pattern. MTP8 has been previously characterized as an Fe and Mn transporter that mediates the loading of Fe and Mn into the embryo (Eroglu et al., 2017). Our staining showed that in almost all *mtp8* seeds, the typical sharp Fe localization appeared diffused (Fig. 5C). Furthermore, in our hands, 11 out of 20 *mtp8* mutant plants showed lighter tones of black (brownish), and 3 out of 20 *mtp8* mutant plants showed no staining at all (i.e., the Fe concentration was below the detection limit). This result indicates that preferential Fe accumulation in the Arabidopsis chalaza is MTP8 dependent. Next, we applied desferoxamine but found no decrease in germination speed of either wild-type or *mtp8* Arabidopsis seeds (data not shown).

Then, we investigated whether the ring-like Fe accumulation corresponds to any known structure in the Arabidopsis seed. A similar structure has recently been identified as the phloem end of the vasculature of the funiculus (Liu et al., 2025). However, this observation was made on developing seeds. There have been no reports on vascular tissue remaining in the mature seeds of Arabidopsis. Due to the similarities between the shapes (i.e., ring-like) and localizations (i.e., at the end of the chalaza), we hypothesize that the Fe-accumulating ring corresponds to the remaining vasculature. To test this hypothesis, we examined the GFP localization in the imbibed mature seeds of SUC2:GFP, a marker used to reveal the symplastic pathway of the phloem (Kalmbach and Helariutta, 2019; Otero and Helariutta, 2016). GFP localized exclusively to the end of the chalaza of the seed, producing a ring-like shape reminiscent of the Fe accumulation (Fig. 5D). This result indicates that the Fe accumulation in the chalaza may follow the vasculature remaining in the seed.

With this new finding, we revisited the unique Fe localization around the squash seeds (Fig. 2F). In most angiosperms, the funiculus attaches to the seed only in the chalaza; however, in the Cucurbitaceae family, a single vasculature extends throughout the outer integument of the seed coat from chalaza to the micropyle in a median plane (Johri et al., 2013) from the rear and the top (Fig. S8A, B). Fe staining patterns matched the expected vascular bundles (Fig. S8C, D). These data indicate that, at least in Arabidopsis and squash, Fe followed vascular tissues in the seeds besides the fruits.

## Discussion

X-ray and histochemistry-based methods highlighted numerous metal hotspots in various plant organs (Eroglu et al., 2019, 2016; Kim et al., 2006). In this study, we examined metal localizations in fruits. Our results show that in fruits, metals including Ca, K, and Fe accumulate in a tissue-specific way. We further show that in Arabidopsis, Fe accumulation in the fruit-seed juncture is mediated by MTP8, possibly determining germination speed in tomato.

### Histochemical staining of freeze-dried and decolorized fruits provides tissue-level information

The histochemical approach to mature fleshy fruits often faces two main obstacles: 1) too much water, limiting the penetration and activity of chemicals, and 2) too many pigments, limiting the visual inspection of the stain. Industrially, fruits have been extensively dried for various purposes. Among drying technologies, vacuum freeze drying appears to be the golden standard for human consumption (Harper and Tappel, 1957). We observed that once freed from water, the fruit slices behaved like regular tissue. After vacuum freeze drying, fixatives could decolorize the fruit. This may represent an advance over the techniques described in the literature. The most common way to circumvent the natural bright colors of fruits like tomato, has been to analyze the sample in earlier developmental stages (Saed Taha et al., 2012; Wang et al., 2014). We propose here that freeze drying, coupled with histochemistry, provides an efficient and quick method to reveal immobile metal hotspots in fruits. Any finding can then be later confirmed with more advanced methods that offer higher resolution and quantification and avoid possible metal re-localization.

### Iron follows the vasculature of fruits and seeds

Fe preferentially accumulates in the vascular tissues of several plant parts. Leaves (Xu et al., 2022), embryos inside the seeds (Kim et al., 2006), and fruits (this study) accumulate Fe in and around their provascular or vascular strands. In mature fruits, vascular Fe may represent the most prominent Fe accumulation site (Fig. 3). However, this accumulation can appear more pronounced than it is, due to clearing off weakly bound Fe in the rest of the fruit during fixation and decolorization. The physiological function of Fe in the fruit vasculature is unclear. Fe deficiency in fruit trees has not been linked with a disease that is specific to fruit vasculature (Àlvarez-Fernàndez et al., 2006). Alternatively, an immobile Fe reservoir may be a “byproduct” of extensive callose production. Callose is required to close the xylem connection in fruits (Knipfer et al., 2015) and may cause the oxidation of a large amount of Fe for its production (Müller et al., 2015). Another possibility is that the main vasculatures of fruits serve as a sink to load Fe into the seeds. In this model, the vasculature may represent a reservoir of excess Fe left over from seed Fe filling, as this process is highly regulated to avoid Fe-mediated oxidative damage in the seeds (Sun et al., 2021). The remaining Fe can then be bound to cell walls that can quickly absorb Fe(III) (Xu et al., 2022). The current study identified the vasculature as the main Fe-accumulating tissue in fruits; however, further investigations to identify the cells in the vasculature that accumulate Fe and into the underlying mechanisms of this process are required to substantiate this supposition.

### Iron in the seed chalaza facilitates germination

A fraction of Fe-loaded vasculature retains in the seeds upon dispersal from the fruit (Fig. 3, 5 and Liu et al., 2023). This Fe reservoir may facilitate germination (Fig. 4). Germination requires softening of the endosperm to allow radical protrusion, a major process that includes cell wall-cutting hydroxyl radicals (Grillet et al., 2013; Muller et al., 2009). A well-known source of hydroxyl radicals derives from the reaction of Fe with H_2_O_2_, referred to as Fenton reaction (Burkitt and Gilbert, 1990b). Therefore, Fe deposits in the chalaza may facilitate germination by generating cell wall-attacking hydroxyl radicals. However, for such a function one would expect Fe to accumulate more towards the micropylar region where the root protrudes. Fe-enriched chalazae are preserved between tomato and Arabidopsis (Fig. 5), but only in tomato perturbation of the Fe deposit by desferoxamine postponed the germination (Fig. 6). This observation implies that the phenotype may depend on the Fe amount in the chalaza. In line with this assumption, tomato seeds that accumulate less Fe (Fig. S4 and Fig. 6) or seeds of species (e.g., Arabidopsis) that accumulate lower concentrations of Fe did not show a clear germination phenotype when treated with desferoxamine. Further research should test whether Fe-mobilizing chelators such as nicotianamine increase germination speed and whether the germination of Arabidopsis Fe over-acccumulating mutants such as *brutus-3* (Hindt et al., 2017) is postponed by desferoxamine.

### Micro-XRF mapping is applicable to investigate nutrient deficiency-related fruit diseases

Micro XRF mapping has been successfully used to examine metal localizations in various plant organs, including leaves, roots, stems, and seeds (Singh et al., 2022). We detected Ca to be preferentially accumulating in the outer part of fruits, especially in the citrus genus (Fig. 1, S10). This is in agreement with previous reports where the highest Ca levels were generally observed in the peel of the fruits (Saure, 2005). Higher Ca in the outer parts of the fruits is probably caused by cell wall thickening. Ca is a major component of the cell wall (Thor, 2019) and outer parts of the fruits usually possess thicker cell walls to maintain integrity (Forlani et al., 2019; Hallett and Sutherland, 2005; Jeffery et al., 2012). When fruits contain insufficient Ca, they may be more prone to infections, as Ca deficiency was reported to cause several diseases, including bitter pit in apples and blossom-end rot in tomatoes, peppers, and watermelons (de Freitas and Mitcham, 2012). Potassium preferentially accumulates in the fleshy parts of the fruits in a conserved manner (Fig. 1 and Ahmed et al., 2024). This is not surprising since K is involved in osmoregulation and sugar accumulation, whereas the fleshy parts of the fruits often accumulate water and sugar (Kumar et al., 2006).

Imaging element distributions of fruits is associated with certain limitations.

Polychromatic excitation with an X-ray tube (Rh in our case) gave a high background due to scattering in the energy range of middle z-elements, decreasing the detection limit for trace elements like Fe, Cu and Zn. We found that in fruits, Fe was under the detection limit for imaging despite the presence of an Fe peak (Fig. S9). Nevertheless, our data showed that micro-XRF can be very useful to image distribution of macro-elements like K and Ca, which can aid in investigating whether specific disease symptoms overlap with perturbations in metal accumulating sites.

## Conclusions

Increasing fruit production should target decreasing pre- and post-harvest losses. To reduce such losses, a better understanding of fruit biology is required. This study extends the basic techniques to localize metals to mature fleshy fruits, contributing to preventing yield loss in fruit production and storage in the long run. Fruits preferentially accumulate Fe in their vascular, K in their fleshy and Ca in their epidermal tissues. Fruits load Fe into the chalazal part of the seeds, which, in *A. thaliana*, is mediated by MTP8. Tomato seed chalaza accumulates a large amount of Fe, which may determine germination speed.

## Supplementary Material

Supplement 1
